# Supplementary figures and images for: Transcriptomic Plasticity Is a Hallmark of Metastatic Pancreatic Cancer
Source: Cancer Res. 2025 Dec 11;86(7):1769–96. doi: 10.1158/0008-5472.CAN-25-1117 (PMC13044532; doi:10.1158/0008-5472.CAN-25-1117)

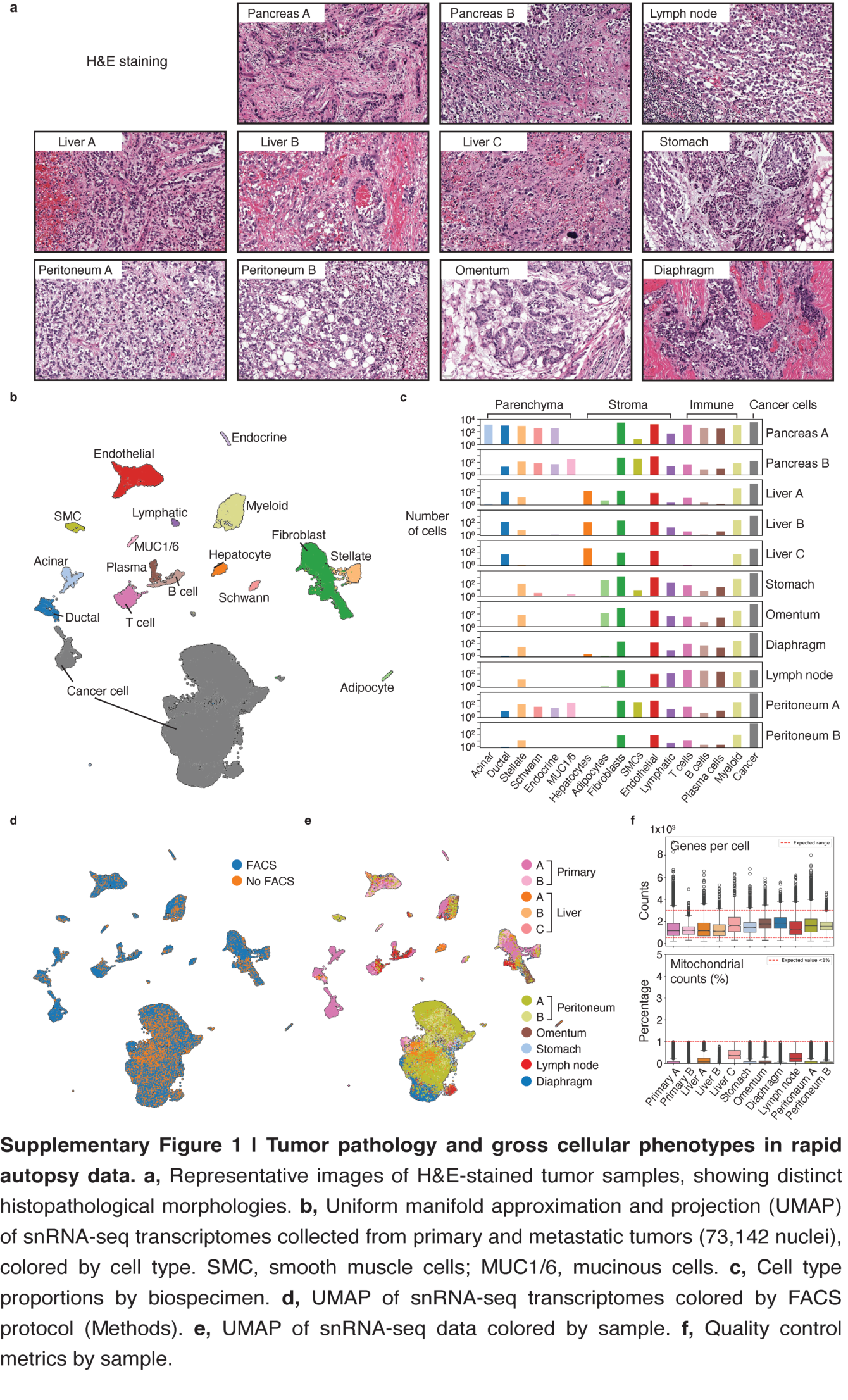

Supplement: Figure S1 — Representative histopathology images and single-nucleus RNA-seq analysis showing cellular diversity, quality control, and phenotypic composition across primary and metastatic PDAC samples [file can-25-1117_figure_s1_suppsf1.png]

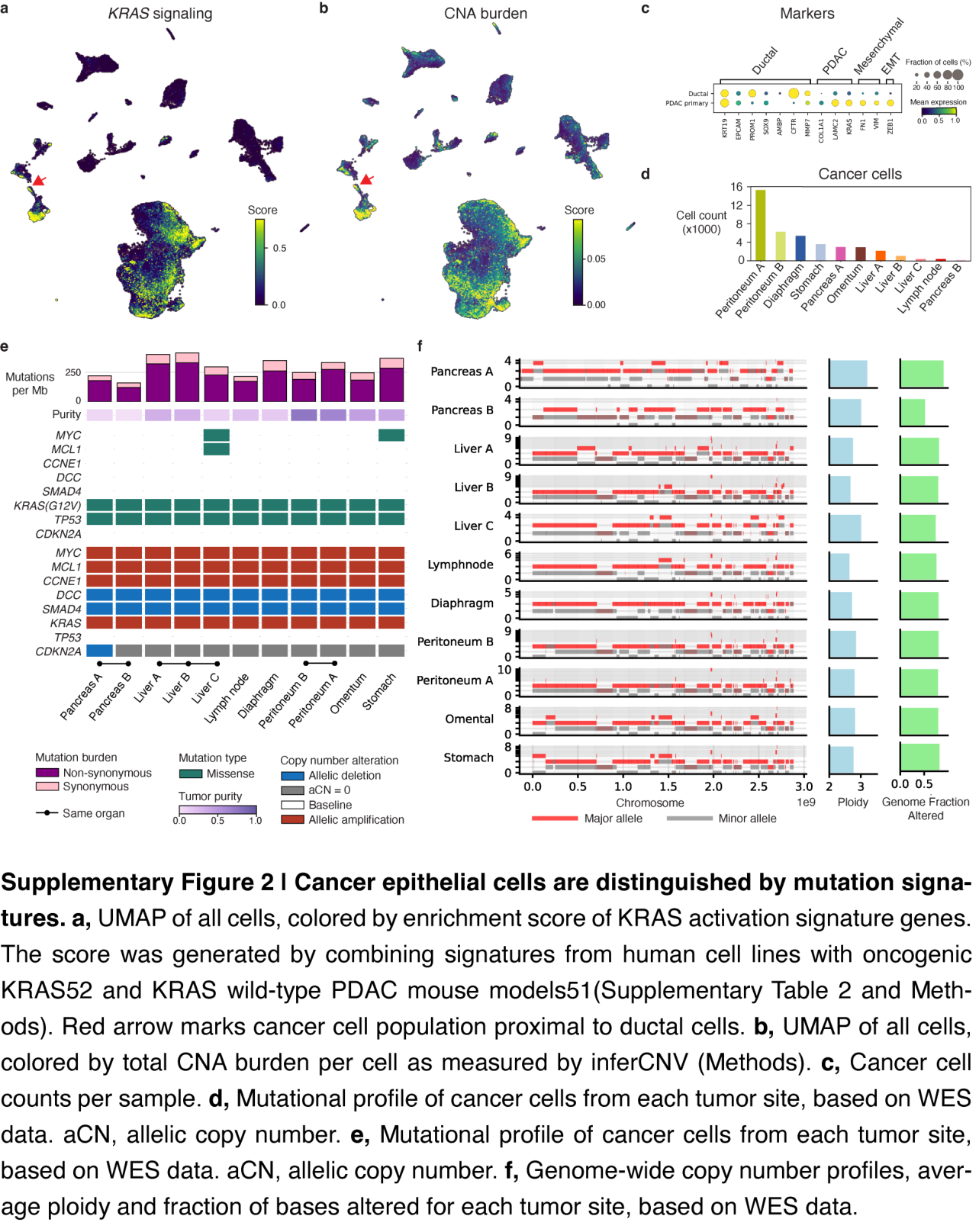

Supplement: Figure S2 — Analysis of PDAC cells showing KRAS pathway activity, copy number alterations, and mutational profiles across metastatic tumor sites [file can-25-1117_figure_s2_suppsf2.png]

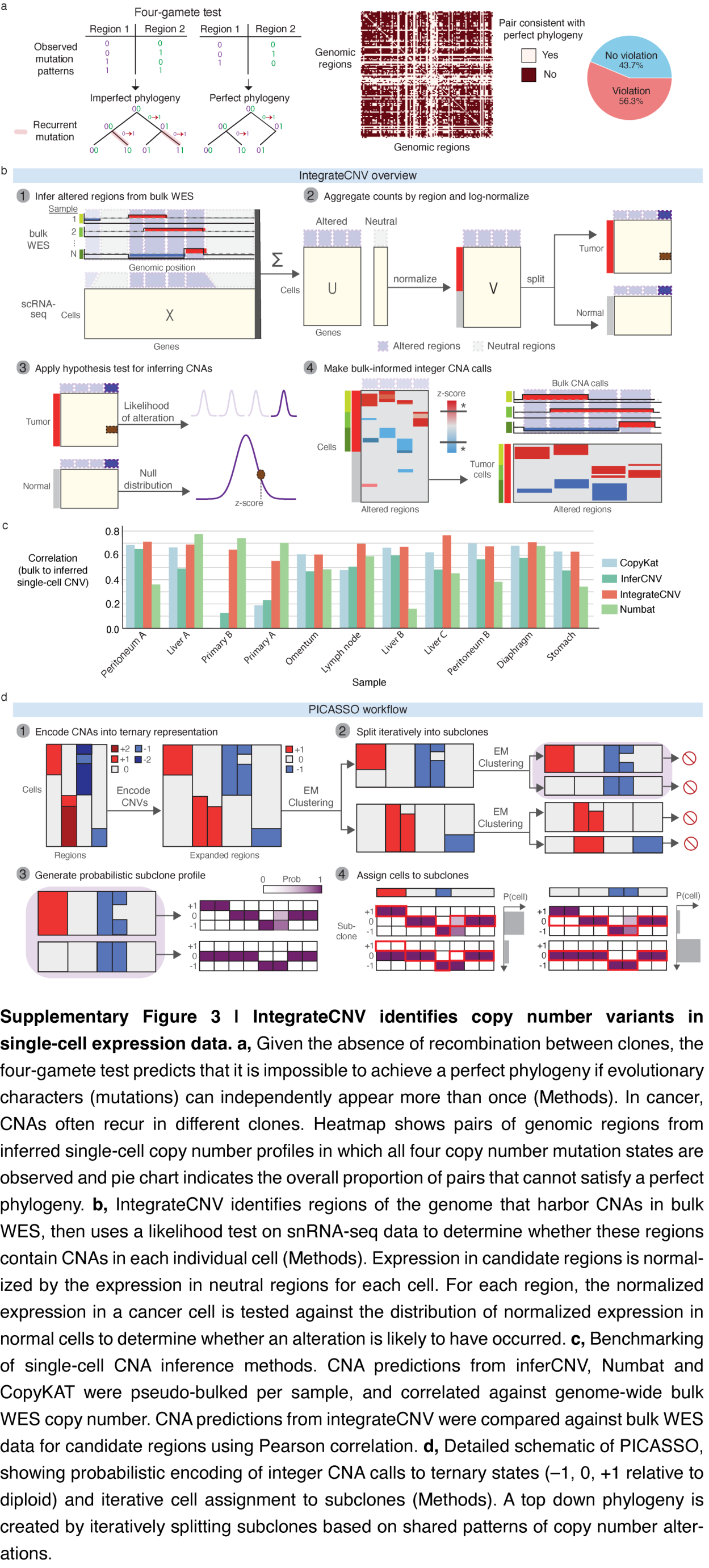

Supplement: Figure S3 — Overview and benchmarking of IntegrateCNV and PICASSO methods for inferring single-cell copy number alterations and reconstructing subclonal phylogenies in metastatic PDAC [file can-25-1117_figure_s3_suppsf3.png]

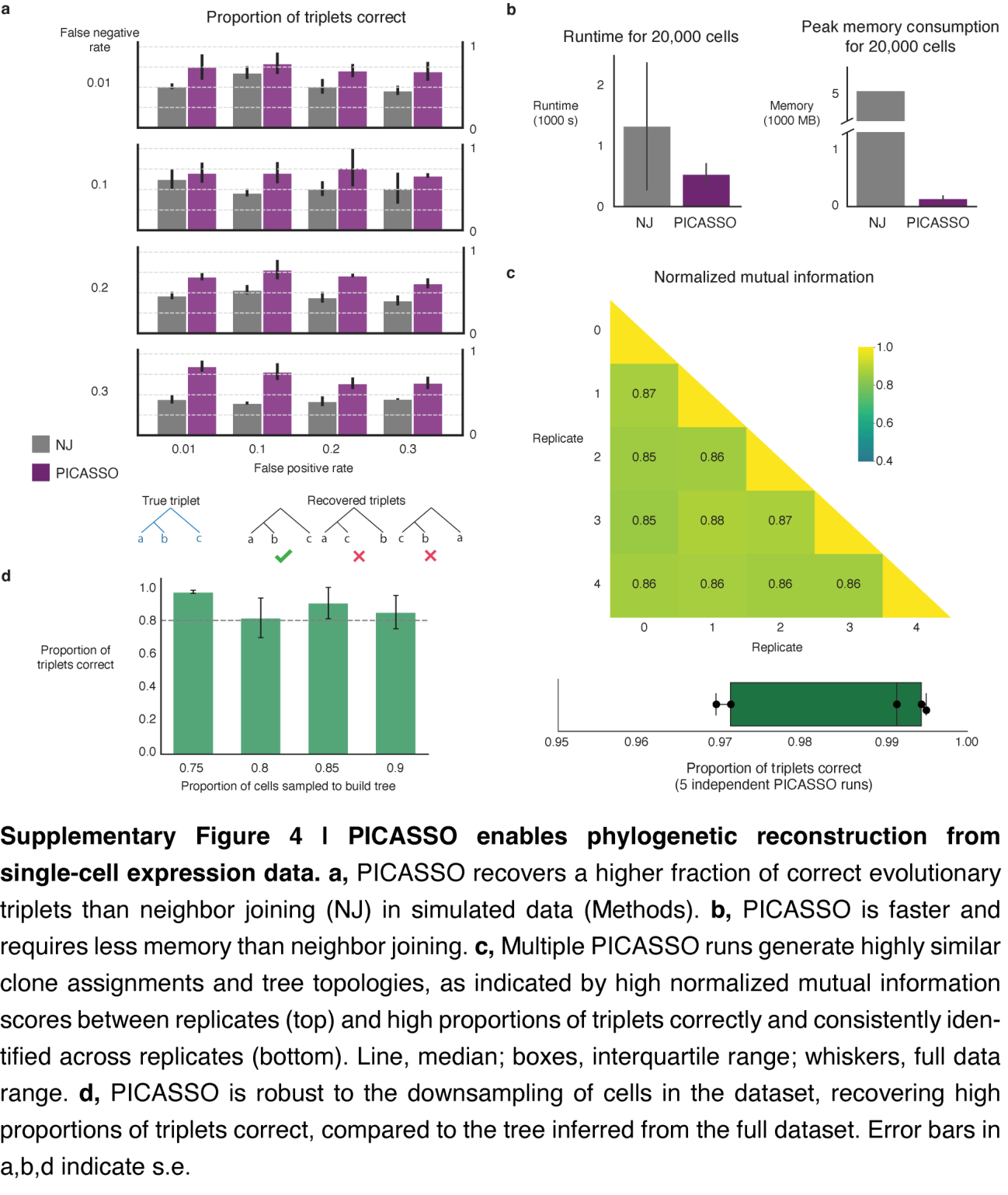

Supplement: Figure S4 — Performance benchmarking of PICASSO showing improved phylogenetic accuracy, computational efficiency, reproducibility, and robustness compared to neighbor-joining approaches [file can-25-1117_figure_s4_suppsf4.png]

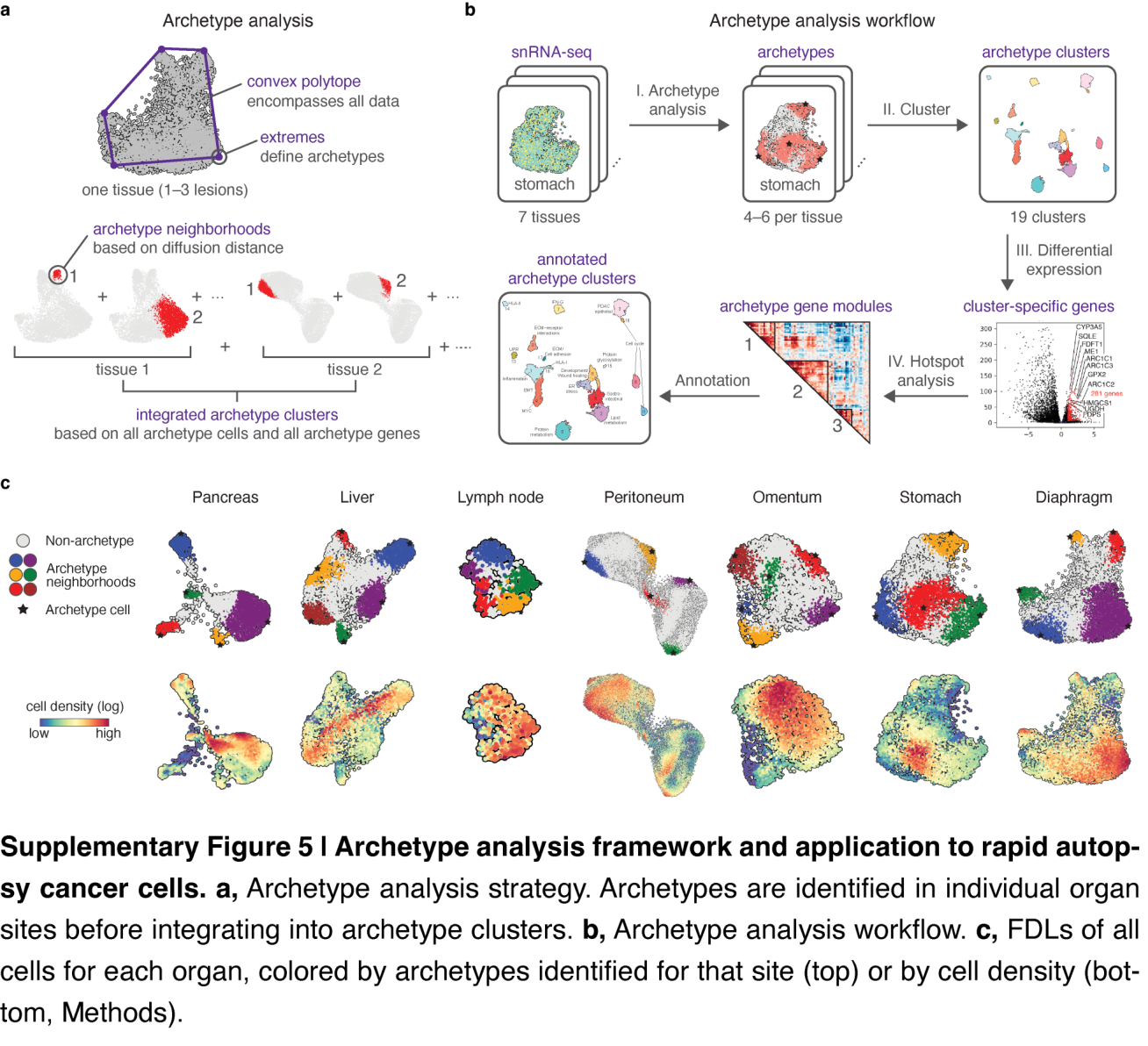

Supplement: Figure S5 — Overview of the archetype analysis framework and its application to single-cell transcriptomes from metastatic organ sites in the rapid autopsy PDAC dataset [file can-25-1117_figure_s5_suppsf5.png]

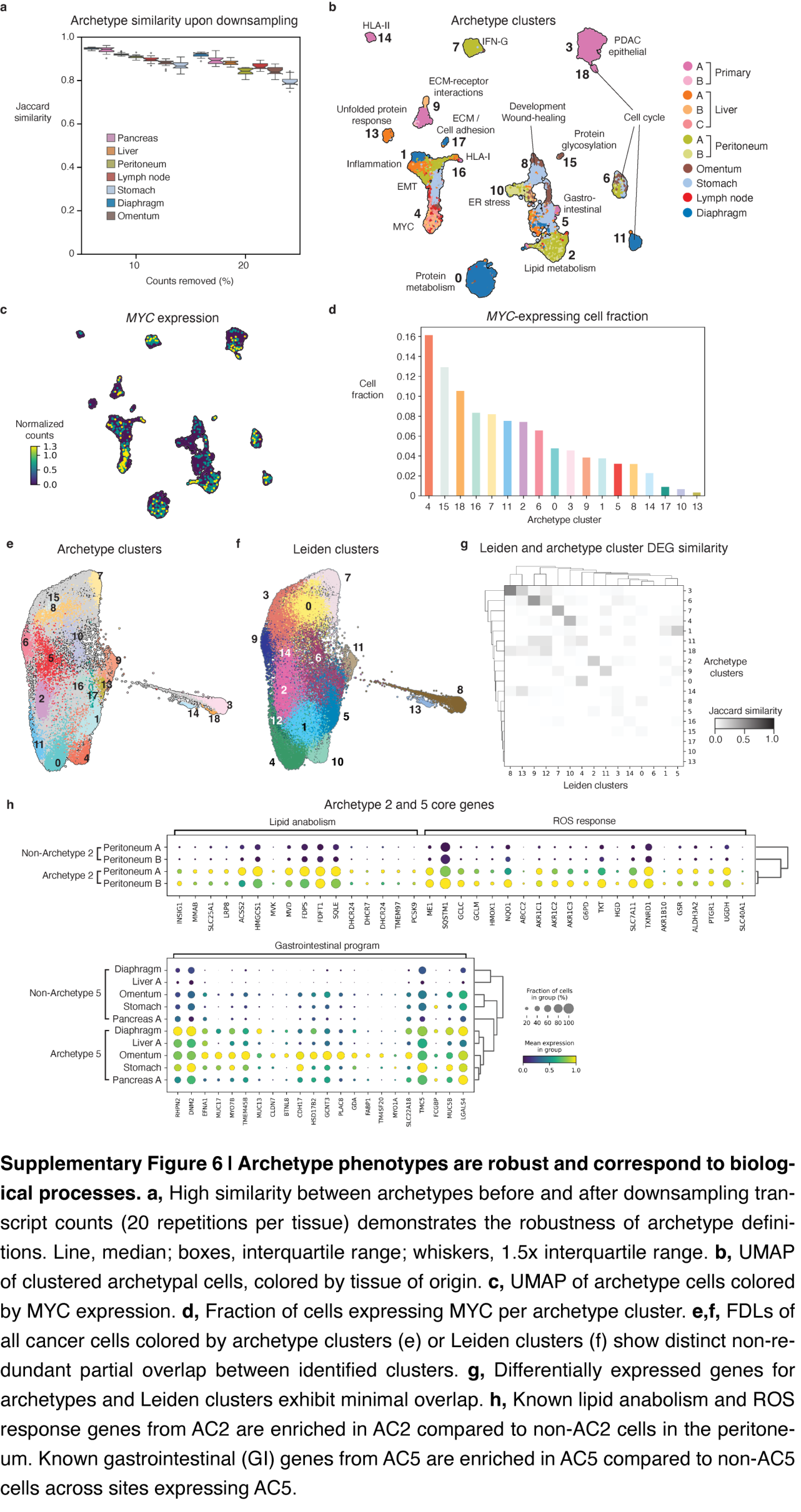

Supplement: Figure S6 — Assessment of archetype robustness and biological significance, showing consistent archetype gene programs across downsampling tests, their association with MYC, lipid metabolism, and gastrointestinal programs, and comparison with clustering analysis [file can-25-1117_figure_s6_suppsf6.png]

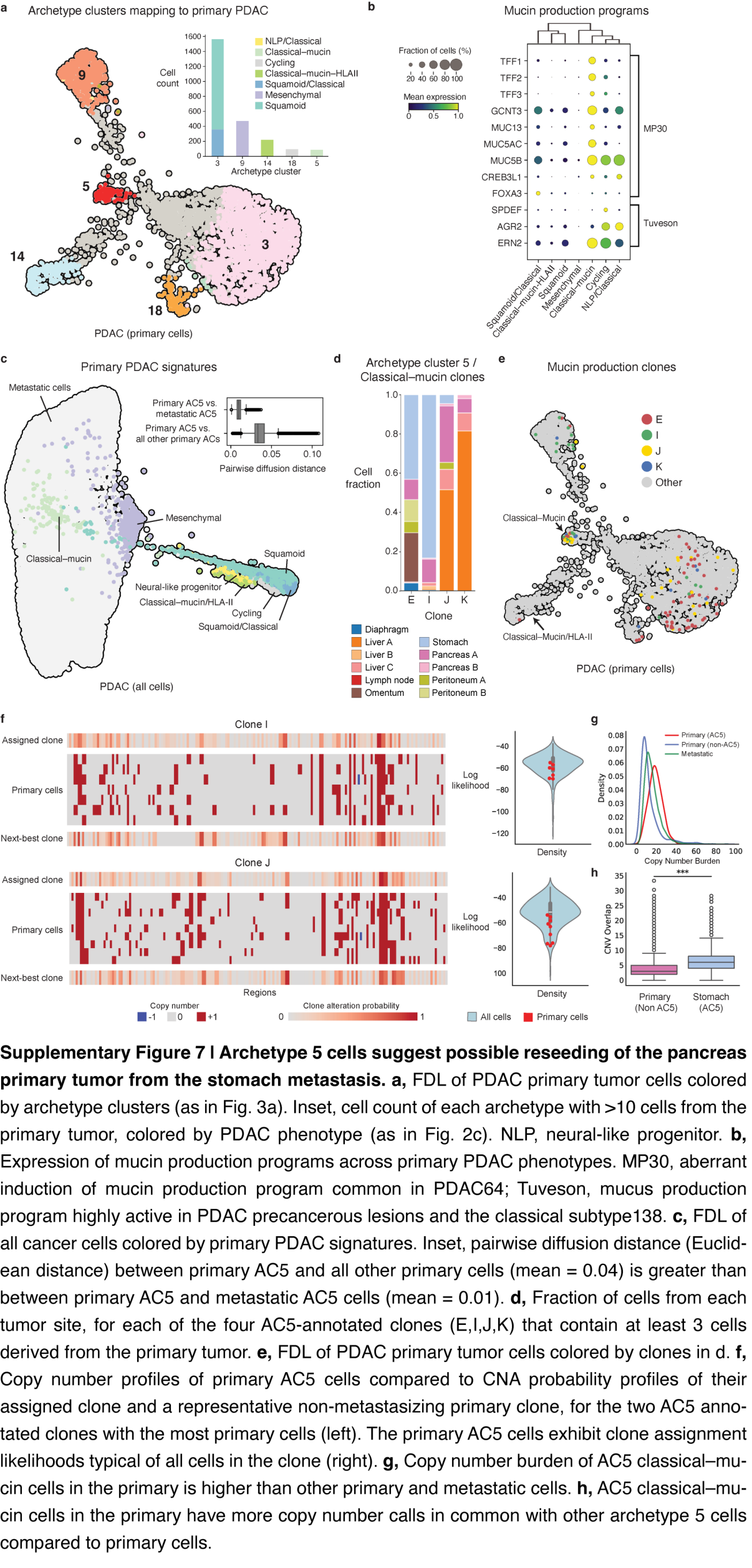

Supplement: Figure S7 — Analysis of archetype 5 cells suggesting possible reseeding of the primary pancreas tumor from the stomach metastasis, integrating expression programs, clonal assignments, and copy-number alterations [file can-25-1117_figure_s7_suppsf7.png]

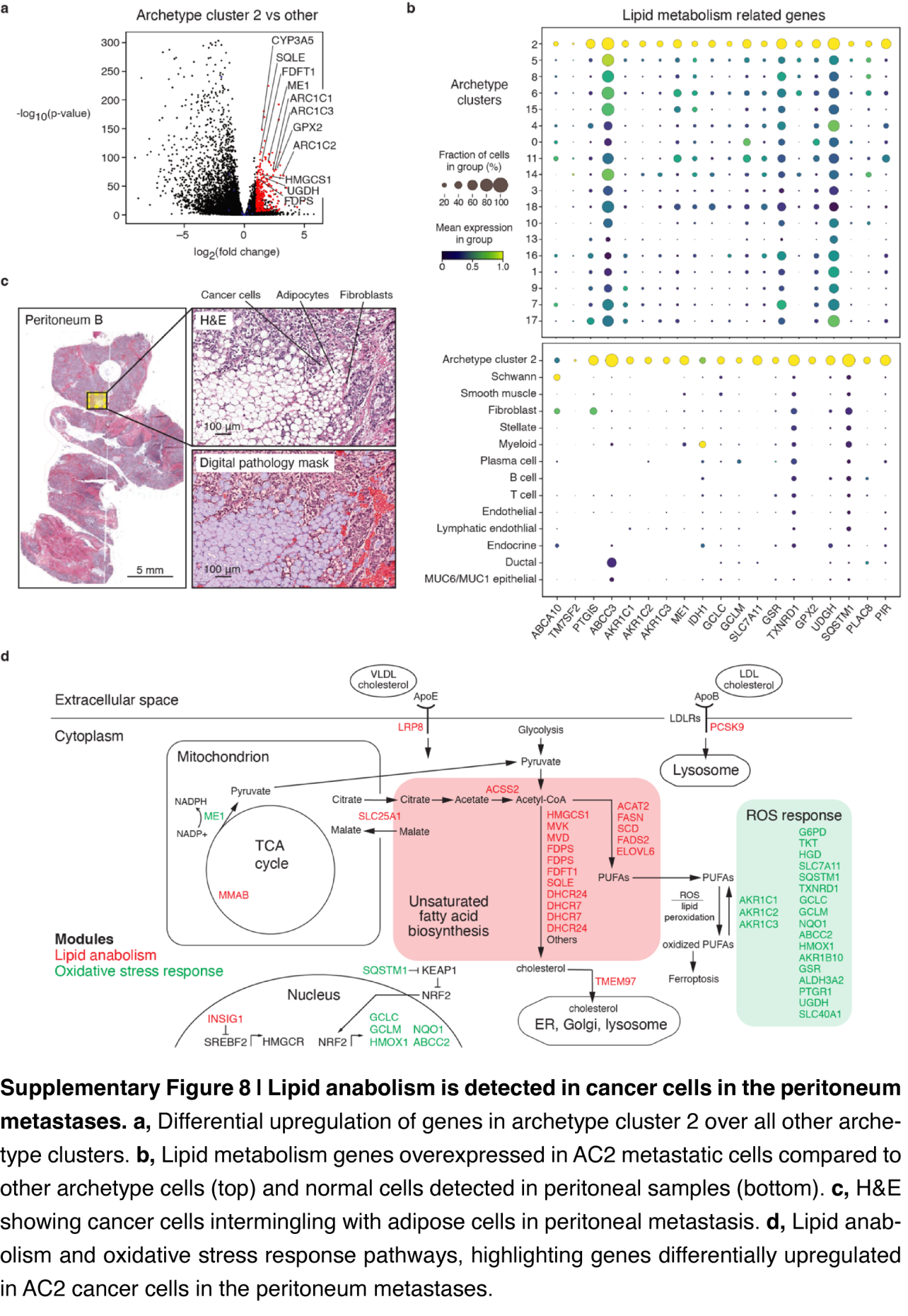

Supplement: Figure S8 — Expression and histological evidence of lipid metabolism in peritoneal metastases, showing up-regulation of lipid anabolism and oxidative stress pathways in archetype 2 cancer cells [file can-25-1117_figure_s8_suppsf8.png]

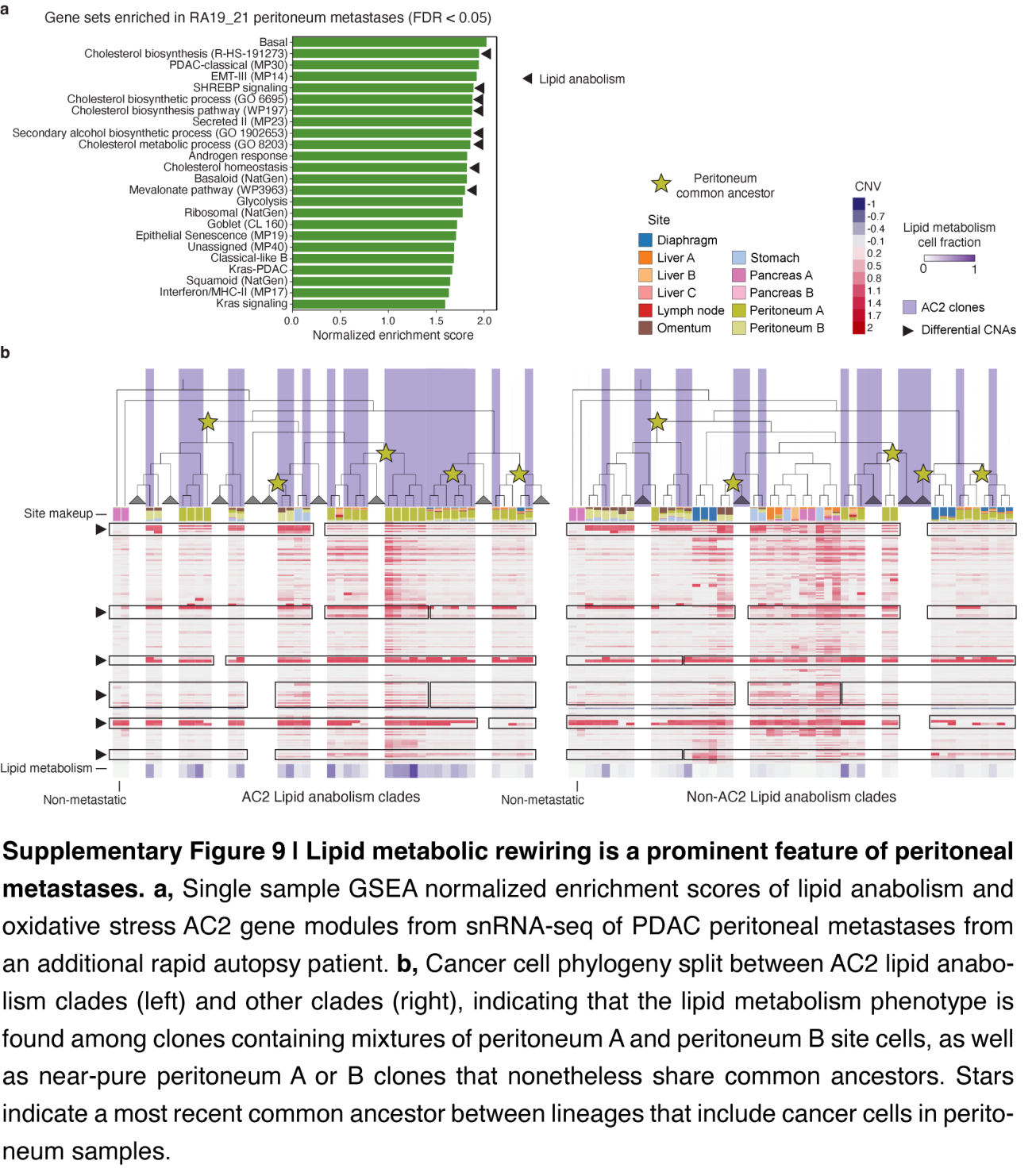

Supplement: Figure S9 — Validation of lipid metabolic rewiring in peritoneal metastases using single-nucleus RNA-seq from an independent rapid-autopsy patient and phylogenetic analysis revealing lipid anabolism up-regulation in peritoneal lesions and archetype 2 clones across distant clades [file can-25-1117_figure_s9_suppsf9.png]

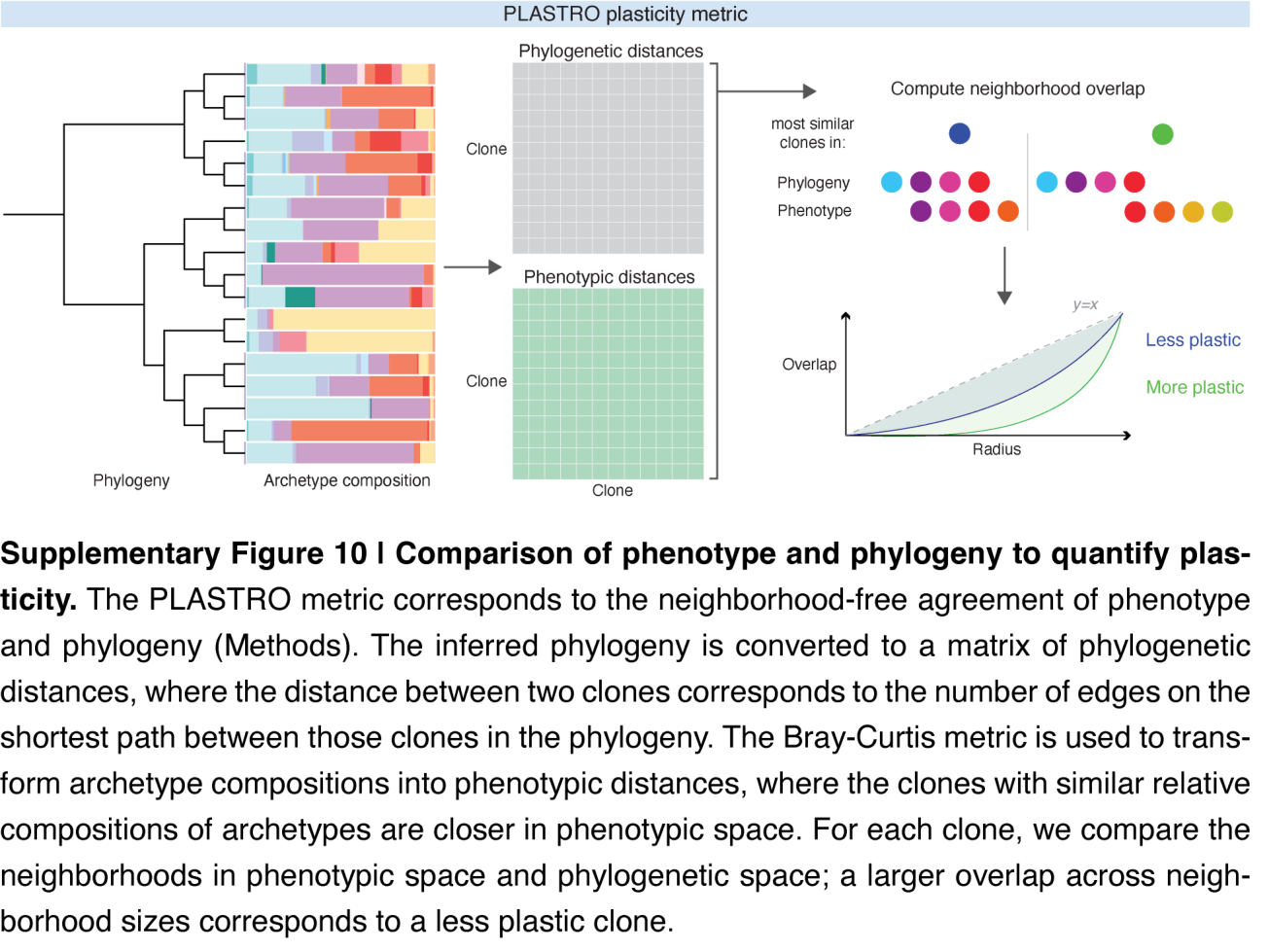

Supplement: Figure S10 — Comparison of phenotypic and phylogenetic relationships using the PLASTRO metric, quantifying the alignment between archetype composition and clonal structure to assess metastatic plasticity [file can-25-1117_figure_s10_suppsf10.png]
